# Integrated Transcriptome and MicroRNAome Analysis Reveals Dynamic Responses Underlying ZmWAK-Mediated Resistance to Head Smut in Maize

**DOI:** 10.3390/jof12100726

**Published:** 2026-09-28

**Authors:** Shengfeng He, Yuanliang Liu, Wenqian Fan, Qianqian Zhang, Suining Deng, Mingliang Xu

**Affiliations:** State Key Laboratory of Maize Bio-Breeding, National Maize Improvement Center, College of Agronomy and Biotechnology, China Agricultural University, Beijing 100193, China; 18813000933@163.com (S.H.); 18813001236@163.com (Y.L.);

**Keywords:** maize head smut, ZmWAK, *Sporisorium reilianum*, transcriptome, microRNAome

## Abstract

The maize wall-associated kinase gene *ZmWAK* confers partial resistance to head smut caused by *Sporisorium reilianum*, yet how it coordinates dynamic immune responses remains poorly understood. Here, we integrated transcriptome and microRNAome analyses of near-isogenic maize lines differing at the *ZmWAK* locus to dissect the regulatory landscape underlying ZmWAK-mediated resistance during mesocotyl infection. We found that ZmWAK was associated with a stage-specific defense trajectory characterized by dynamic shifts in phytohormone networks, intermediate cellular reprogramming, and late-stage cell wall remodeling. A conserved extracellular defense signature was maintained through sustained activation of apoplastic oxidoreductase-associated processes. Meanwhile, ZmWAK-associated miRNAs were preferentially reprogrammed during peak pathogen proliferation, and their predicted targets were enriched in phenylpropanoid metabolism, lignin biosynthesis, secondary metabolism, and apoplastic oxidoreductase activity. These patterns suggest that transcriptional and post-transcriptional responses converge on extracellular redox and cell wall-associated defense processes. Together, our findings define a dynamic, multilayered regulatory landscape associated with ZmWAK-mediated quantitative resistance and provide insights into how resistance loci coordinate temporal immune responses during pathogen infection.

## 1. Introduction

Maize head smut is a devastating soil-borne disease caused by the biotrophic fungus *Sporisorium reilianum*. The pathogen invades maize seedlings primarily through the roots or coleoptiles and establishes a prolonged biotrophic interaction within host tissues. As maize transitions from vegetative to reproductive growth, *S. reilianum* undergoes extensive proliferation in tassel and ear tissues, replacing developing reproductive structures with masses of dark teliospores and ultimately causing severe yield losses or complete crop failure [1,2,3,4].

The maize wall-associated kinase gene *ZmWAK*, which is absent from some elite maize inbred lines such as Huangzao4 and Chang7-2, is the functional gene underlying the major-effect quantitative resistance locus *qHSR1*, reducing maize head smut incidence by ~25% [5,6]. Through marker-assisted selection, *qHSR1* has been extensively utilized in maize breeding programs to enhance head smut resistance, contributing to the improvement of the Chinese Tangsipingtou germplasm and the development of resistant elite inbred lines and varieties [7]. Recent studies have identified ZmWAK as an important regulator coordinating plant growth and immunity. Under non-infected conditions, ZmWAK promotes the expression of genes associated with cell growth, whereas *S. reilianum* infection triggers a transcriptional transition toward defense responses, characterized by the activation of genes involved in chitinase activity and reactive oxygen species (ROS) production [8]. More recently, the ZmWAK-ZmSnRK1α2-ZmWRKY signaling module was shown to suppress pathogen proliferation by restricting nutrient transport to the apoplast [9]. Despite these advances, how ZmWAK shapes genome-wide transcriptional responses throughout pathogen invasion remains poorly understood, particularly during the early stages of mesocotyl colonization. Furthermore, whether post-transcriptional regulatory processes are associated with ZmWAK-mediated resistance and how they relate to transcriptional responses during infection remain largely unknown.

MicroRNAs (miRNAs) are endogenous small non-coding RNAs, typically 18–24 nucleotides in length, that meticulously fine-tune gene expression through post-transcriptional mechanisms, including target mRNA cleavage and translational inhibition [10,11]. Although initially identified as regulators of plant growth, development, and phase transitions [12,13,14,15], miRNAs are now recognized as critical modulators of plant immune responses [16,17,18,19,20]. In *Arabidopsis*, for example, miR164 attenuates pathogen-induced cell death by targeting the transcription factor *NAC4* [21]. In rice, the miR172a-*SNB* regulatory module confers broad-spectrum disease resistance by promoting lignin biosynthesis through activation of the downstream *MYB30* pathway [22]. Similarly, maize miR167 contributes to resistance against chlorotic mottle virus by regulating the *ZmARF3/30* module and promoting ROS accumulation [23]. Together, these studies establish miRNAs as important regulators of plant-pathogen interactions. However, whether miRNA responses are associated with ZmWAK-mediated resistance to head smut, and how these responses relate to transcriptional reprogramming during *S. reilianum* infection, remains unknown.

To address these questions, we performed an integrated transcriptome and miRNAome analysis of mesocotyl tissues from two near-isogenic lines (NILs), Huangzao4 (HZ4) and Huangzao4R (HZ4R), which differ primarily at the *ZmWAK* locus. Samples were collected under both *S. reilianum*-inoculated and mock-treated conditions across three infection stages (5, 7, and 9 days after sowing). By integrating temporal transcriptomic and miRNA expression profiles, we characterized the multilayered regulatory landscape associated with ZmWAK-mediated resistance. Our study reveals that ZmWAK is associated with stage-specific transcriptional reprogramming and miRNA-associated responses, providing insights into how quantitative resistance loci coordinate dynamic immune responses during pathogen infection.

## 2. Materials and Methods

### 2.1. Plant Materials and Treatments

The near-isogenic lines (NILs) used in this study, Huangzao4 (HZ4) and Huangzao4R (HZ4R), which share 99.65% genetic background identity, were described previously [8]. HZ4 lacks the *ZmWAK* locus and is highly susceptible to head smut, whereas HZ4R was generated by crossing HZ4 as the recurrent parent with the highly resistant line Ji1037 as the donor parent, followed by nine generations of backcrossing and subsequent selfing (BC_9_F_1_). Consequently, HZ4R carries the resistant *ZmWAK* allele and exhibits enhanced resistance to *S. reilianum* infection.

Seeds were surface-sterilized, soaked in sterile water for 2 h, and sown at a depth of 3.5 cm in pots (9 cm in diameter and 7 cm in height) containing soil that had been autoclaved at 121 °C for 20 min before use. For pathogen inoculation, seeds were planted in sterilized soil supplemented with 0.2% (*w*/*w*) *S. reilianum* teliospores, whereas seeds planted in sterilized soil without teliospores served as mock-treated controls. All plants were grown in a controlled greenhouse under a 16 h light/8 h dark photoperiod until sampling.

### 2.2. RNA Extraction and cDNA Synthesis

Total RNA was extracted using the EASYspin Plant RNA Kit (Aidlab, Beijing, China, catalog no. RN09) according to the manufacturer’s instructions. First-strand cDNA was synthesized using TRUEscript RT MasterMix (OneStep gDNA Removal) (Aidlab, Beijing, China, catalog no. PC7002), in accordance with the manufacturer’s protocols.

Mesocotyl tissues were collected at 5, 7, and 9 days after sowing (DAS). Seedlings were carefully removed from the soil and gently rinsed with sterile water to remove adhering soil particles. After excess surface moisture was removed using sterile absorbent paper, mesocotyl tissues were excised with a sterile blade, and immediately frozen in liquid nitrogen. Frozen mesocotyl samples were ground into a fine homogeneous powder using an autoclaved mortar and pestle. The homogenized tissue was promptly transferred into 2 mL RNase-free microcentrifuge tubes for subsequent RNA extraction.

### 2.3. S. reilianum Biomass Quantification

The relative biomass of *S. reilianum* in maize mesocotyl tissues was quantified by quantitative reverse transcription PCR (qRT-PCR) at 5, 7, and 9 DAS. The fungal actin gene (*SrActin*) was used as a pathogen-specific marker, and the maize *ZmGAPDH* gene was used as an internal control. qRT-PCR was performed using ChamQ Blue Universal SYBR qPCR Master Mix (Vazyme, Nanjing, China, catalog no. Q312) following the manufacturer’s instructions. Each assay included three biological replicates, with two technical replicates analyzed per biological replicate. Relative fungal biomass was calculated using the $2^{{-\Delta \Delta C}_{T}}$ method [24].

### 2.4. Transcriptome and MiRNAome Sequencing

Mesocotyl samples collected from HZ4 and HZ4R plants under mock-treated and *S. reilianum*-inoculated conditions at 5, 7, and 9 DAS were subjected to mRNA and miRNA sequencing. Total RNA extraction, library construction, and sequencing were performed by Novogene Co., Ltd. (Beijing, China). following the manufacturer’s protocols. mRNA sequencing libraries were prepared for paired-end sequencing using the Illumina PE150 platform, whereas small RNA libraries were prepared for single-end sequencing using the Illumina SE50 platform.

### 2.5. Data Processing and Differential Expression Analysis

Following quality filtering, clean reads were aligned to the maize B73 reference genome (v5). Identification of known miRNAs was carried out via sequence alignment against the miRBase database. Because a mature miRNA can be derived from multiple precursor transcripts, a single representative name was assigned to each mature miRNA to eliminate redundancy. Novel miRNAs were predicted using an integrated approach combining the outputs of miREvo (v1.1) and miRDeep2 (v0.1.3) algorithms.

Expression levels were normalized as transcripts per million (TPM) for miRNAs and fragments per kilobase of transcript per million mapped reads (FPKM) for mRNAs. Differential expression analysis was performed using the DESeq2 (v1.42.0) package [25], and differentially expressed genes (DEGs) or miRNAs (DEMs) were identified with thresholds of |log_2_(fold change)| > 1 and false discovery rate (FDR) < 0.05.

MiRNA targets were predicted using the psRNATarget website (https://www.zhaolab.org/psRNATarget/, accessed on 10 September 2026) with a prediction threshold of 3.5 and default parameters for all other settings.

### 2.6. GO Enrichment Analysis

Gene Ontology (GO) enrichment analysis of DEGs was performed using the clusterProfiler (v4.8.1) package [26]. The most significantly enriched GO terms were selected for visualization. Enrichment bar-dot plots were generated using the SRplot online visualization platform [27].

## 3. Results

### 3.1. Infection Dynamics of S. reilianum and Establishment of Temporal Sampling Stages

Previous studies showed that *S. reilianum* inoculation induces slight mesocotyl cell necrosis in HZ4R at 7 days after sowing (DAS), representing an early cytological response associated with ZmWAK-mediated resistance, whereas HZ4 remains asymptomatic at the same stage [8]. To capture the temporal progression of *S. reilianum* infection, we selected 7 DAS as the central sampling point, corresponding to the initial emergence of necrotic lesions in HZ4R. Mesocotyl tissues were additionally collected at 5 DAS (shortly after seedling emergence) and 9 DAS (two-leaf stage) to monitor pathogen proliferation and host responses (Figure 1A).

Quantification of fungal biomass revealed distinct infection dynamics between the two NILs. At 5 DAS, fungal colonization was relatively limited and showed no significant difference between HZ4 and HZ4R. However, by 7 DAS, fungal biomass increased dramatically in the susceptible HZ4 line, exhibiting an ~35-fold increase compared with the earlier stage, and remained at a high level at 9 DAS. In contrast, fungal proliferation was strongly restricted in HZ4R, showing only a modest increase at 7 DAS and reaching approximately two-fifths of the maximum fungal biomass detected in HZ4 by 9 DAS (Figure 1B). These contrasting trajectories are consistent with restriction of *S. reilianum* proliferation in the resistant HZ4R background during the transition from early colonization to extensive fungal growth.

Based on these infection dynamics, we operationally defined 5, 7, and 9 DAS as early, intermediate, and late sampling stages, respectively. Mesocotyl samples collected at these three stages were subjected to integrated transcriptome and miRNAome sequencing.

### 3.2. Identification of ZmWAK-Associated S. reilianum-Responsive Transcriptional Changes

To characterize transcriptional responses associated with ZmWAK-mediated resistance, we performed four pairwise comparisons at each sampling stage. RC (HZ4R, control (mock)-treated) versus HC (HZ4, mock-treated) and RS (HZ4R, *S. reilianum*-inoculated) versus HS (HZ4, *S. reilianum*-inoculated) captured genotype-associated expression differences under mock and infected conditions, respectively. HS versus HC and RS versus RC captured infection-responsive expression changes within HZ4 and HZ4R, respectively. At 5, 7, and 9 DAS, 707, 3932, and 1383 differentially expressed genes (DEGs), respectively, were identified between HZ4R and HZ4 under mock conditions (RC vs. HC), whereas 890, 2189, and 1383 DEGs were detected between the two genotypes under infection (RS vs. HS). In response to *S. reilianum*, 890, 2396, and 3484 DEGs were detected in HZ4 (HS vs. HC), compared with 807, 1376, and 693 DEGs in HZ4R (RS vs. RC) (Figure 2A). Thus, the magnitude of transcriptional differences varied substantially across genotype, treatment, and sampling stage. Notably, the largest number of DEGs at 7 DAS occurred between HZ4R and HZ4 under mock conditions (RC vs. HC; 3932 DEGs), indicating substantial basal transcriptional differences between the two NILs at this stage.

To identify infection-responsive genes whose expression was also associated with the HZ4/HZ4R genetic contrast, we next integrated the genotype and infection comparisons. Genotype-associated DEGs were defined as genes differentially expressed between HZ4R and HZ4 under either mock or infected conditions (RC vs. HC ∪ RS vs. HS), whereas *S. reilianum*-responsive (*SRR*) DEGs were defined as genes responsive to infection in either HZ4 or HZ4R (HS vs. HC ∪ RS vs. RC). The intersection of these two sets, [(RC vs. HC ∪ RS vs. HS) ∩ (HS vs. HC ∪ RS vs. RC)], identified *SRR* genes whose expression was also associated with the genetic difference between HZ4 and HZ4R (Figure 2B). We refer to these genes hereafter as ZmWAK-associated *SRR* DEGs. This intersection-based analysis was used to define candidate expression patterns associated with ZmWAK-mediated resistance, while the direction and temporal structure of their responses were further examined by expression-pattern analyses below.

Using this approach, we identified 421, 2067, and 1029 ZmWAK-associated *SRR* DEGs at 5, 7, and 9 DAS, respectively (Figure 2C–E; Tables S1–S3). The number of identified DEGs was highest at 7 DAS, approximately fivefold that at 5 DAS, indicating that the intermediate sampling stage was associated with the most extensive transcriptional differences captured by this analytical framework. This peak coincided temporally with the emergence of localized mesocotyl necrosis in HZ4R and with the divergence in fungal colonization between HZ4 and HZ4R. The number of ZmWAK-associated *SRR* DEGs subsequently decreased at 9 DAS, indicating that the composition and magnitude of the transcriptional response changed substantially over the course of infection.

### 3.3. Stage-Specific Transcriptional Reprogramming Associated with ZmWAK During S. reilianum Infection

To resolve the temporal and directional expression patterns of ZmWAK-associated *SRR* DEGs, we performed hierarchical clustering followed by Gene Ontology (GO) enrichment analysis. The DEGs identified at 5 DAS were grouped into six expression clusters, whereas those identified at 7 and 9 DAS were each grouped into four clusters (Figure 3A–F). These clusters revealed distinct stage-dependent expression patterns and functional enrichments during *S. reilianum* infection.

At 5 DAS, ZmWAK-associated *SRR* DEGs were primarily enriched in endogenous phytohormone signaling pathways, including auxin (Figure 3B, cluster 1), brassinosteroid (Figure 3B, cluster 3), and ethylene responses (Figure 3B, cluster 5), suggesting that hormone-related regulatory networks are linked to the early establishment of resistance-associated responses. Additionally, processes related to transcriptional regulation (Figure 3B, cluster 2) and ubiquitination-dependent protein degradation (Figure 3B, cluster 6) were also specifically enriched at this stage, reflecting the rapid transcriptome reprogramming and protein turnover required for early defense activation. At 7 DAS, the enriched processes shifted toward cell cycle regulation and microtubule-associated functions (Figure 3D, cluster 4), as well as the abscisic acid (Figure 3D, cluster 1) and cytokinin (Figure 3D, cluster 2) signaling pathways. These patterns suggest that ZmWAK-associated resistance involves extensive changes in cellular organization and hormone-associated processes during the intermediate infection stage. By 9 DAS, ZmWAK-associated *SRR* DEGs were predominantly enriched in the jasmonic acid signaling pathway (Figure 3F, cluster 3) and cell wall-associated processes (Figure 3F, cluster 2), which include polysaccharide and xyloglucan metabolism, indicating a potential transition toward cell wall remodeling and structural reinforcement during the later infection stage. In addition, DNA repair-related processes, such as the DNA damage response (Figure 3F, cluster 1), were specifically enriched at this stage, suggesting the involvement of genome maintenance responses under prolonged pathogen challenge. Collectively, these results reveal a stage-dependent transcriptional trajectory associated with ZmWAK, characterized by dynamic shifts in phytohormone networks throughout the infection, alongside intermediate cellular reprogramming and late-stage cell wall remodeling.

Beyond these stage-specific biological processes, molecular function analysis unveiled a persistent defense-related signature throughout the infection period. Across all three stages, heme binding, tetrapyrrole binding, and monooxygenase activity were among the most significantly enriched molecular functions, accompanied by stage-specific enrichment of iron ion binding at 5 and 7 DAS, and oxidoreductase activity at 7 and 9 DAS. These patterns indicate a sustained association of metal-dependent redox processes with ZmWAK-mediated defense responses, likely reflecting the importance of redox regulation during prolonged host–pathogen interactions. Furthermore, a consistent enrichment of genes encoding defense-related proteins localized to the extracellular region was observed across all three stages. The sustained transcriptional activation of these apoplastic defense components suggests that extracellular immunity represents a crucial aspect of ZmWAK-associated resistance as the infection progresses.

### 3.4. Temporal Dynamics of the miRNAs Landscape in Maize Mesocotyls

To characterize the temporal dynamics of miRNA regulation during *S. reilianum* infection, we profiled miRNA populations in maize mesocotyl tissues across three infection stages. A total of 240 miRNAs were detected across the three sampling stages, revealing a dynamic yet largely conserved miRNA repertoire. Among these, 96 miRNAs were detected consistently at all three stages, whereas the total number of detected miRNAs showed only moderate variation, with 162, 142, and 167 miRNAs detected at 5, 7, and 9 DAS, respectively. These included 121 known and 41 novel miRNAs at 5 DAS, 120 known and 22 novel miRNAs at 7 DAS, and 130 known and 37 novel miRNAs at 9 DAS (Figure 4A,B; Tables S4–S6). Novel miRNAs were defined based on the presence of canonical hairpin structures in their precursor sequences (Tables S4–S6). The conserved and stage-specific miRNA populations identified in maize mesocotyls provide a foundation for investigating their potential roles in regulating developmental and stress-responsive processes during infection.

The identified known miRNAs were classified into 30 families, with miR156, miR159, miR164, miR166, miR167, miR169, and miR171 among the most abundant families (Figure 4C, Table S7). The predominant accumulation of these conserved miRNA families suggests that fundamental regulatory networks are maintained during *S. reilianum* infection. Several of these families have previously been implicated in the regulation of plant development, hormone signaling, stress responses, and immunity. For instance, miR164 and miR167 participate in auxin-associated regulatory pathways and stress-responsive processes, whereas miR156 and miR159 contribute to developmental phase transitions [16]. The enrichment of these conserved miRNA families indicates that endogenous miRNA networks associated with growth and stress adaptation are active in maize mesocotyl tissues during pathogen challenge.

Analysis of miRNA length distribution revealed distinct patterns between known and novel miRNAs, with 21-nt species predominating among known miRNAs and 24-nt species being relatively enriched among novel miRNAs (Figure 4D). In plants, 21-nt miRNAs generally function through canonical post-transcriptional regulation, including target mRNA cleavage or translational repression, whereas 24-nt small RNAs are commonly associated with epigenetic regulation through RNA-directed DNA methylation pathways [28,29]. Notably, 22-nt miRNAs represented the second most abundant size class in both known and novel miRNA populations, consistent with their reported capacity to trigger secondary small RNA production in plants [30]. Collectively, these distinct size distributions reveal a diverse miRNA regulatory landscape in maize mesocotyl tissues and suggest the involvement of multiple layers of miRNA-mediated regulation during the host–pathogen interaction.

### 3.5. Temporal Reprogramming of ZmWAK-Associated miRNAs During S. reilianum Infection

To examine miRNA responses associated with ZmWAK-mediated resistance, we analyzed the miRNAome using the same comparative framework applied to the transcriptome. In contrast to the extensive transcriptional changes observed at the mRNA level, relatively few differentially expressed miRNAs (DEMs) were detected across the comparisons. At 5, 7, and 9 DAS, respectively, 8, 5, and 6 DEMs were identified between HZ4R and HZ4 under mock conditions (RC vs. HC), whereas 6, 7, and 3 DEMs were detected between the two genotypes under infection (RS vs. HS). The infection-responsive miRNA profiles also differed between the two genetic backgrounds. *S. reilianum* infection was associated with 4, 10, and 4 DEMs in HZ4 (HS vs. HC), compared with 0, 3, and 1 DEMs in HZ4R (RS vs. RC) at 5, 7, and 9 DAS, respectively (Figure 5A–C). Thus, under the criteria used here, fewer infection-responsive miRNAs were detected in HZ4R than in HZ4.

We next integrated the genotype and infection comparisons using the same framework applied to the transcriptome to identify ZmWAK-associated *S. reilianum*-responsive (*SRR*) DEMs. Across the three sampling stages, a total of 13 ZmWAK-associated *SRR* DEMs were identified. Among these, three DEMs (miR528a-5p, miR162-3p, and novel-33) were detected at 5 DAS; nine (miR528a-5p, miR528a-3p, miR408b-5p, miR408a, miR398a-5p, miR397a-5p, miR11970-5p, miR168a-3p, and miR1156k-3p) at 7 DAS; and three (miR408b-5p, miR172b-5p, and novel-66) at 9 DAS (Figure 5A–D). The largest number of ZmWAK-associated *SRR* DEMs was detected at 7 DAS, coinciding with the pronounced divergence in fungal colonization between HZ4 and HZ4R and with the extensive transcriptional changes observed at this stage. Examination of expression directionality revealed distinct miRNA response patterns at 7 DAS. Six of the nine identified DEMs exhibited infection-induced upregulation in the susceptible HZ4 line, whereas their expression remained unchanged or reduced in the resistant HZ4R line. In parallel, the remaining three DEMs showed decreased expression in HZ4R but unchanged or increased expression in HZ4 following infection (Figure 5D). Thus, most of the identified DEMs showed contrasting infection-responsive patterns between the two genetic backgrounds, with infection-associated miRNA accumulation generally attenuated in HZ4R. These patterns are consistent with a potential negative association between the accumulation of these miRNAs and the resistant phenotype, although their functional contributions to ZmWAK-mediated resistance remain to be determined. Among the identified DEMs, miR528a-5p was detected at both 5 and 7 DAS and exhibited a stage-dependent expression pattern. Following *S. reilianum* infection, miR528a-5p was induced in HZ4R at 5 DAS but displayed a contrasting response at 7 DAS (Figure 5D). This temporal transition suggests that miR528a-5p undergoes dynamic regulation during infection and may participate in stage-specific regulatory processes associated with ZmWAK-dependent responses. The distinct temporal behavior of miR528a-5p highlights the complexity of miRNA-mediated regulation during the progression of maize-*S. reilianum* interactions.

### 3.6. Predicted Targets of ZmWAK-Associated miRNA Converge on Extracellular Redox and Cell Wall-Associated Processes

Putative targets of the identified ZmWAK-associated *SRR* DEMs were predicted using psRNATarget, yielding 142 expressed target genes in maize mesocotyl tissues (Table S7). Given that the 7 DAS stage exhibited the highest number of ZmWAK-associated *SRR* DEMs and the greatest infection-responsive transcriptional changes, we focused on this stage to investigate the potential regulatory relationships between these miRNAs and their predicted targets. As plant miRNAs regulate target genes through either transcript cleavage or translational repression, their regulatory effects are not necessarily reflected by a simple inverse correlation between miRNA abundance and target transcript levels. Consistent with this complexity, only a subset of predicted targets displayed clear opposite expression patterns relative to their corresponding miRNAs (Figure 5E). Nevertheless, functional enrichment analysis of these targets revealed a coordinated post-transcriptional regulatory signature associated with ZmWAK-dependent resistance responses.

GO enrichment analysis showed that the predicted targets of ZmWAK-associated *SRR* DEMs were significantly associated with phenylpropanoid metabolism, lignin biosynthesis, and secondary metabolic pathways. At the molecular function level, these targets were enriched in copper ion binding and oxidoreductase activity toward diphenols, while their encoded proteins were preferentially localized to the apoplast and extracellular region (Figure 5F). These functional characteristics are consistent with the known roles of apoplastic copper-dependent oxidoreductases, including laccase-like proteins, in oxidative phenylpropanoid metabolism and cell wall-associated defense processes. The contrasting accumulation patterns of the corresponding miRNAs between HZ4 and HZ4R suggest that these miRNA–target relationships may contribute to differences in extracellular defense-associated responses during infection, although their functional effects remain to be experimentally validated (Figure 5F).

Notably, the functional characteristics of these miRNA targets showed strong convergence with the persistent transcriptomic defense signature identified in ZmWAK-associated *SRR* DEGs, particularly with respect to extracellular localization and oxidoreductase-associated activities (Figure 3). This integrative analysis suggests that transcriptional and post-transcriptional regulatory layers converge on apoplastic redox-related and cell wall-associated defense processes during *S. reilianum* infection. The prominence of these miRNA-associated patterns at 7 DAS further raises the possibility that miRNA-mediated regulation contributes to the temporal modulation of extracellular defense-associated processes during the intermediate stage of infection.

## 4. Discussion

Quantitative resistance provides durable protection against pathogens by integrating multilayered defense responses while avoiding the fitness penalties associated with constitutive immune activation [31,32,33]. However, how resistance loci achieve such balanced regulation remains poorly understood. Here, integrated transcriptome and miRNAome analyses of near-isogenic maize lines differing primarily at the *ZmWAK* locus reveal that ZmWAK-mediated resistance to head smut involves a dynamic regulatory architecture rather than a single defense pathway. Our findings suggest that *ZmWAK* contributes to the coordination of transcriptional and post-transcriptional responses that integrate immune priming, cellular adjustment, redox regulation, and structural defense during pathogen infection.

A major finding of this study is that ZmWAK-associated responses exhibit distinct temporal patterns during *S. reilianum* infection. This dynamic trajectory is characterized by stage-dependent shifts in phytohormone-associated processes, intermediate cellular reprogramming, and later enrichment of cell wall-associated functions. At 7 DAS, DEGs associated with the cell cycle were broadly down-regulated in the resistant line HZ4R, suggesting a transient shift away from growth-associated cellular programs during the intermediate stage of infection. Such a pattern is consistent with the broader concept that plants dynamically balance growth and immunity during pathogen challenge [34,35], although its direct contribution to ZmWAK-mediated resistance remains to be established. Beyond these stage-specific changes, a persistent extracellular redox-associated signature was evident throughout the infection period, including enrichment of metal-binding and oxidoreductase-related functions together with genes associated with the apoplast and extracellular region. The coexistence of temporally dynamic intracellular responses and a persistent extracellular redox signature suggests that ZmWAK-mediated resistance is associated with both stage-specific regulatory adjustments and sustained apoplastic defense-related processes during prolonged *S. reilianum* infection.

Alongside transcriptional regulation, our miRNAome analysis revealed a potential post-transcriptional regulatory layer associated with ZmWAK-dependent resistance. ZmWAK-associated *SRR* miRNAs were most prominent at 7 DAS, and their predicted targets were enriched in phenylpropanoid and lignin-related metabolism, apoplastic localization, and oxidoreductase-associated functions. These features converged with the persistent extracellular redox signature identified at the transcriptome level, suggesting that transcriptional and potential post-transcriptional responses converge on apoplastic redox and cell wall-associated processes during infection. Although the functional effects of individual miRNA–target relationships remain to be established, their temporally structured expression patterns raise the possibility that miRNAs contribute to modulating these extracellular defense-associated processes over the course of infection. Together, the coexistence of stage-specific transcriptional responses, a persistent extracellular redox signature, and temporally structured miRNA changes highlights the multilayered nature of ZmWAK-mediated quantitative resistance. Such temporal and regulatory flexibility may represent an important feature of quantitative disease resistance.

A further feature of the temporal response was the marked difference in the magnitude of infection-associated molecular changes between the two genetic backgrounds. At the intermediate and late sampling stages, *S. reilianum* infection was associated with substantially more DEGs in susceptible HZ4 than in resistant HZ4R. A similar, although more limited, pattern was observed at the miRNA level, with more infection-responsive DEMs detected in HZ4 than in HZ4R. These differences coincided with the pronounced divergence in fungal colonization between the two lines, particularly at 7 and 9 DAS, making it difficult to distinguish genotype-associated regulation from secondary responses to differences in pathogen abundance. Nevertheless, the comparatively restricted molecular changes in HZ4R are consistent with a more constrained host response accompanying resistance, whereas the broader reprogramming in HZ4 may reflect both host responses to infection and the consequences of greater pathogen proliferation. Hierarchical clustering further resolved the direction and temporal structure of these expression changes, revealing stage-specific patterns associated with hormone signaling, cellular processes, and cell wall-related functions. Thus, rather than demonstrating that ZmWAK directly buffers pathogen-driven transcriptional disruption, our data support an association between ZmWAK-mediated resistance, restricted pathogen proliferation, and a more constrained molecular response during the later stages of infection.

Previous studies established that ZmWAK restricts head smut development through defense activation and nutrient regulation mediated by the ZmWAK-ZmSnRK1α2-ZmWRKY signaling module [9]. Our findings extend this mechanistic framework by providing a genome-wide, temporal view of the molecular responses associated with ZmWAK-mediated resistance. Across successive stages of *S. reilianum* infection, these responses encompassed stage-specific shifts in hormone-associated and cellular processes, a persistent extracellular redox signature, later enrichment of cell wall-associated functions, and a temporally structured miRNA response whose predicted targets converged on apoplastic redox and phenylpropanoid-related processes. Together, these observations suggest that the activity of a major quantitative resistance locus is accompanied by coordinated changes across multiple regulatory layers rather than by sustained activation of a single defense pathway. This multilayered and temporally dynamic response provides a broader framework for understanding how major resistance loci are associated with quantitative immunity during pathogen infection.

Several questions arising from this study will require further experimental investigation. In particular, the functions of the identified ZmWAK-associated miRNAs and their predicted targets should be tested genetically and biochemically to determine whether they directly contribute to head smut resistance and to validate the proposed miRNA–target relationships. Targeted phytohormone measurements and metabolomic analyses will also be valuable for determining whether the transcriptional signatures identified here are accompanied by corresponding changes in hormone abundance, redox metabolism, and cell wall-related metabolites. In addition, because fungal colonization diverged substantially between HZ4 and HZ4R at the intermediate and late sampling stages, future experiments that more closely match pathogen abundance or resolve host responses at higher temporal resolution will help distinguish genotype-associated regulation from secondary responses to differences in pathogen proliferation. Together, such studies should clarify how ZmWAK-associated transcriptional and post-transcriptional responses are mechanistically connected and determine which components directly contribute to durable quantitative resistance.

## Figures and Tables

**Figure 1:**
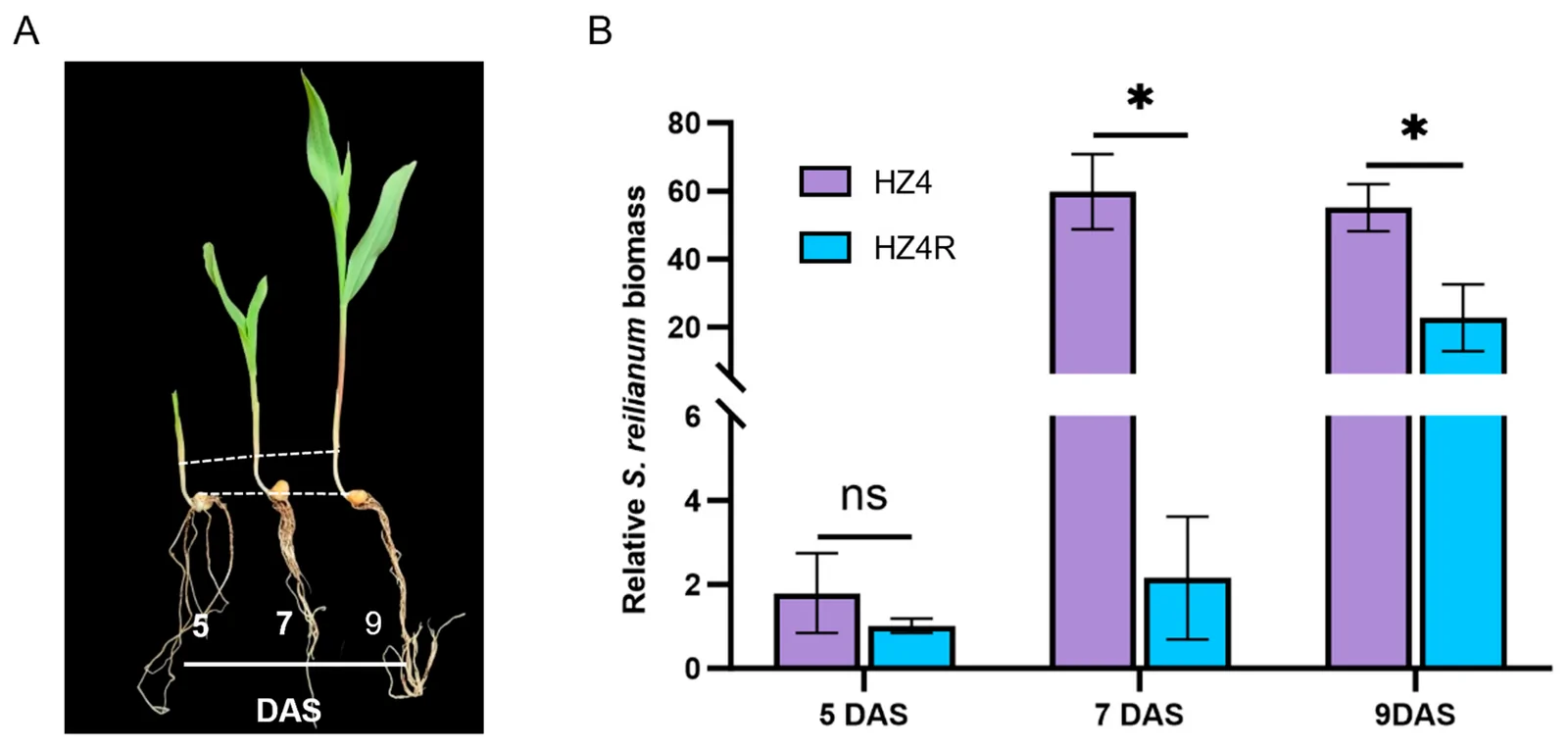
Temporal dynamics of *S. reilianum* colonization in HZ4 and HZ4R. (**A**) Schematic diagram of the experimental design and sampling strategy. Mesocotyl samples were collected from HZ4 (susceptible) and HZ4R (resistant) at 5, 7, and 9 days after sowing (DAS) under both non-inoculated and *S. reilianum*-inoculated conditions. The representative photographs shown in the schematic are from HZ4. (**B**) Relative fungal biomass in mesocotyls of HZ4 and HZ4R at the indicated time points. Values represent means ± SD of three biological replicates. *, *p* < 0.05; ns, not significant.

**Figure 2:**
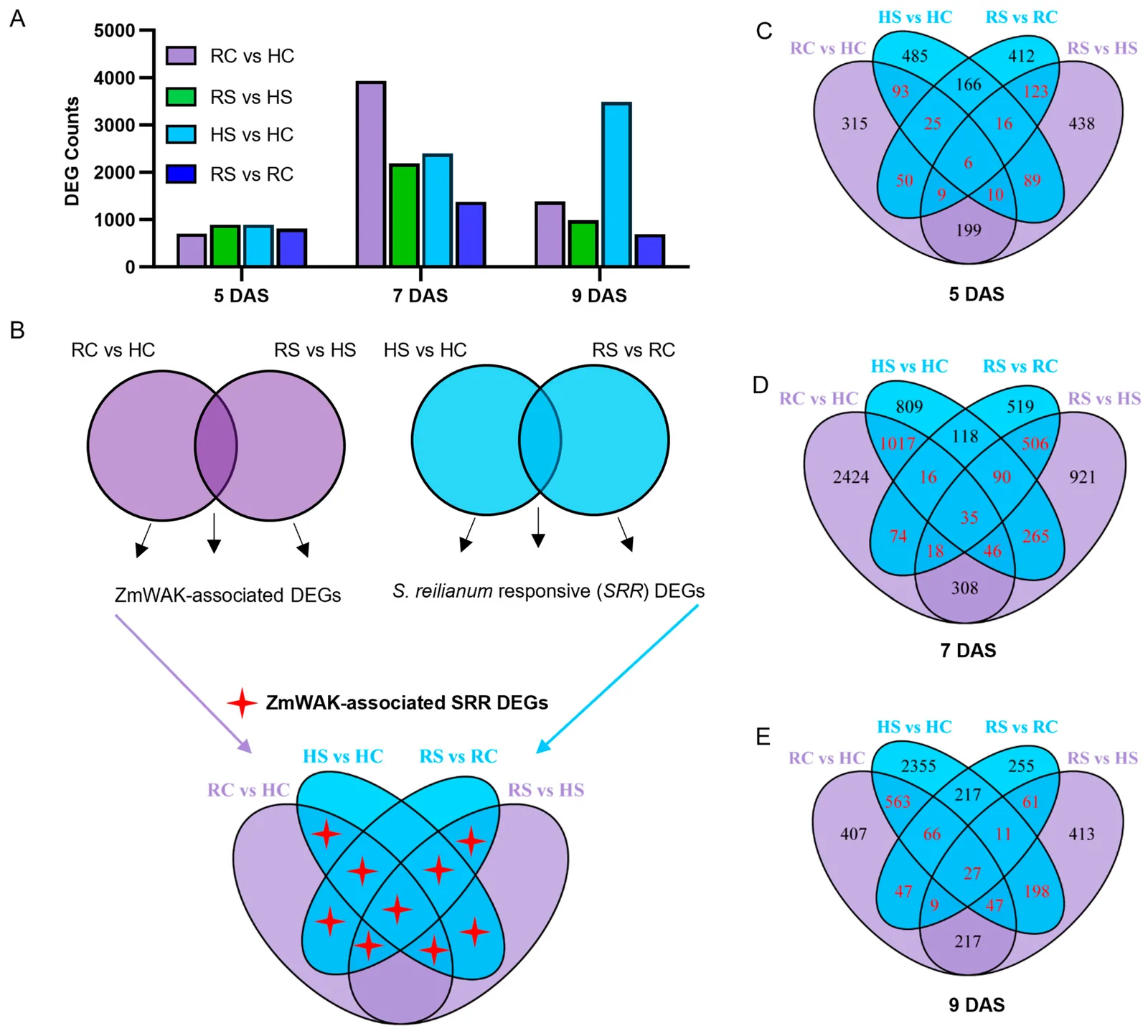
Identification of ZmWAK-associated *S. reilianum*-responsive (*SRR*) genes during infection. (**A**) Numbers of differentially expressed genes (DEGs) identified in four pairwise comparisons across three time points. HC: HZ4 under control (mock) treatment, RC: HZ4R under mock treatment, HS: HZ4 inoculated with *S. reilianum*, RS: HZ4R inoculated with *S. reilianum*. (**B**) Schematic diagram illustrating the strategy used to identify ZmWAK-associated *S. reilianum*-responsive (*SRR*) genes. Genotype-associated DEGs were defined from RC versus HC and RS versus HS, whereas *SRR* DEGs were defined from HS versus HC and RS versus RC. Their intersection was designated as ZmWAK-associated *SRR* DEGs and is indicated by red asterisks. (**C**–**E**) Venn diagrams showing the four DEG comparisons at 5 (**C**), 7 (**D**), and 9 (**E**) DAS. Red numbers indicate ZmWAK-associated *SRR* DEGs.

**Figure 3:**
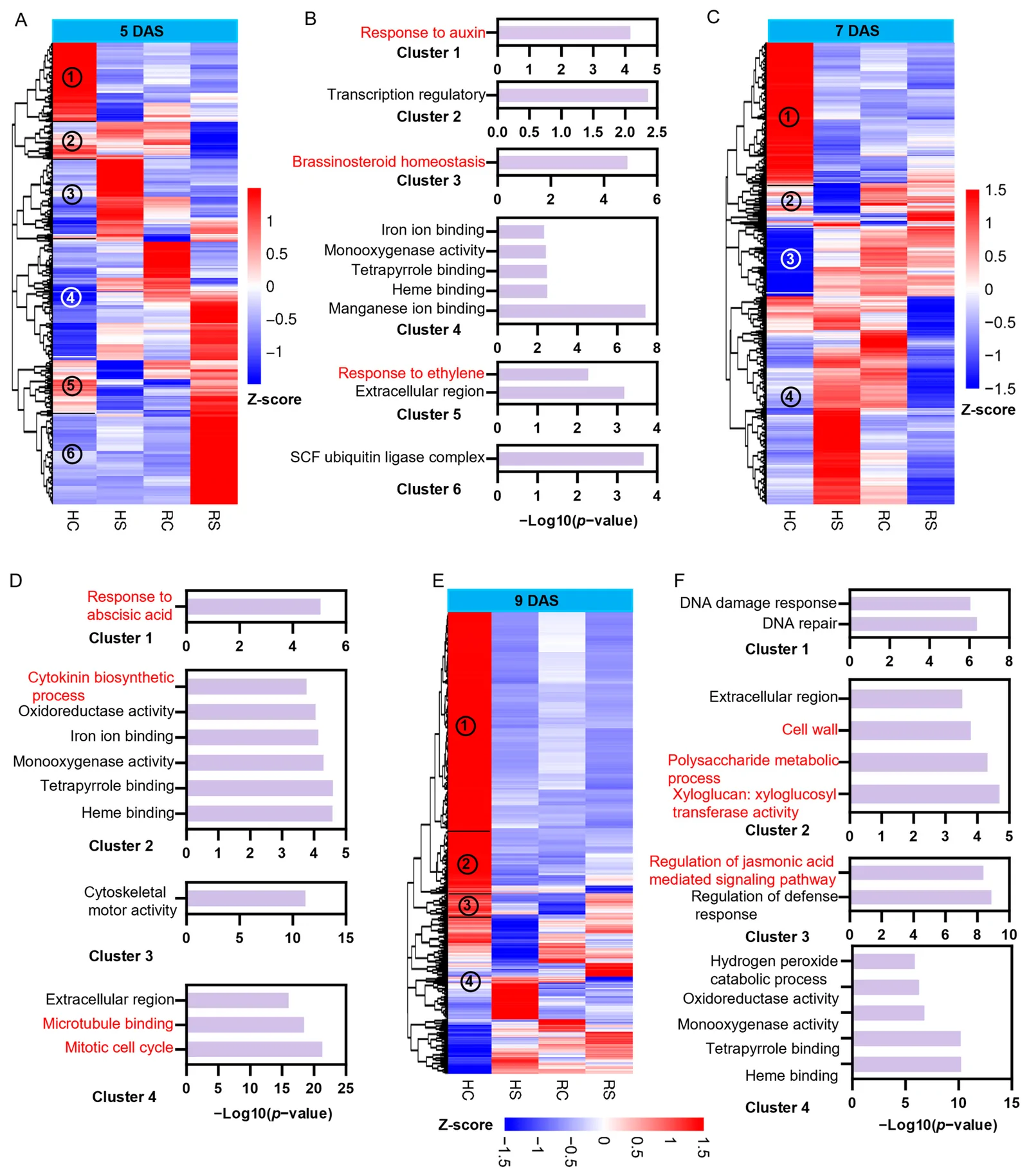
Stage-specific expression patterns and functional enrichment of ZmWAK-associated *SRR* DEGs. (**A**,**C**,**E**) Hierarchical clustering of ZmWAK-associated *SRR* DEGs identified at 5 (**A**), 7 (**C**), and 9 (**E**) DAS, respectively. The heatmaps display the relative expression levels (Z-scores) across HC, HS, RC, and RS samples. (**B**,**D**,**F**) Representative Gene Ontology (GO) terms enriched in the corresponding expression clusters at 5 (**B**), 7 (**D**), and 9 (**F**) DAS. Stage-specifically enriched terms are indicated in red. HC, HZ4 under mock treatment; HS, HZ4 inoculated with *S. reilianum*; RC, HZ4R under mock treatment; RS, HZ4R inoculated with *S. reilianum*.

**Figure 4:**
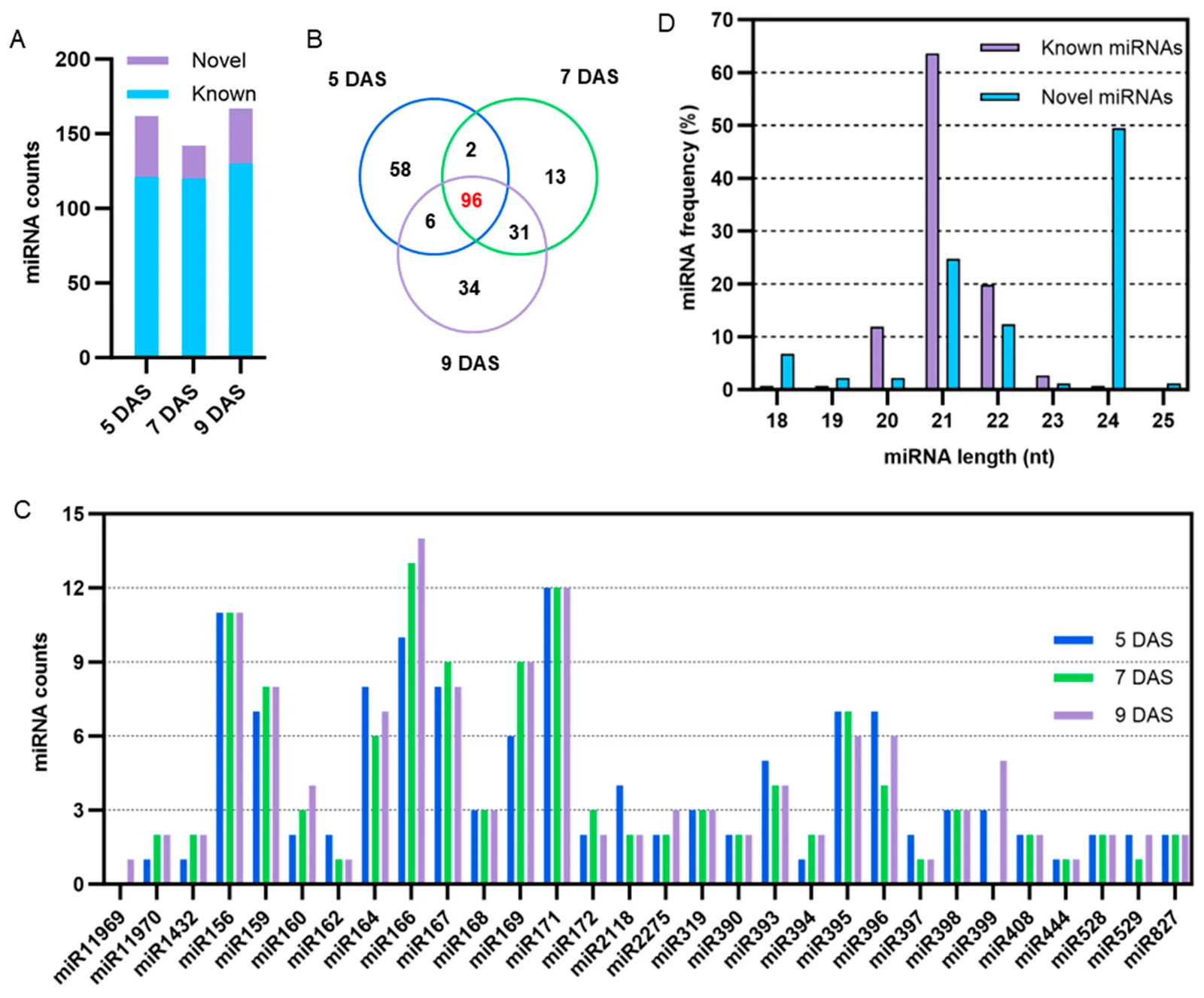
Temporal profiles of miRNAs detected in maize mesocotyls during *S. reilianum* infection. (**A**) Numbers of known and novel miRNAs detected at 5, 7, and 9 DAS. (**B**) Venn diagram showing shared and stage-specific miRNAs detected across the three sampling stages. (**C**) Family distribution of known miRNAs detected at 5, 7, and 9 DAS. (**D**) Length distribution of known and novel miRNAs. The 21-nt class predominated among known miRNAs, whereas the 24-nt class predominated among novel miRNAs.

**Figure 5:**
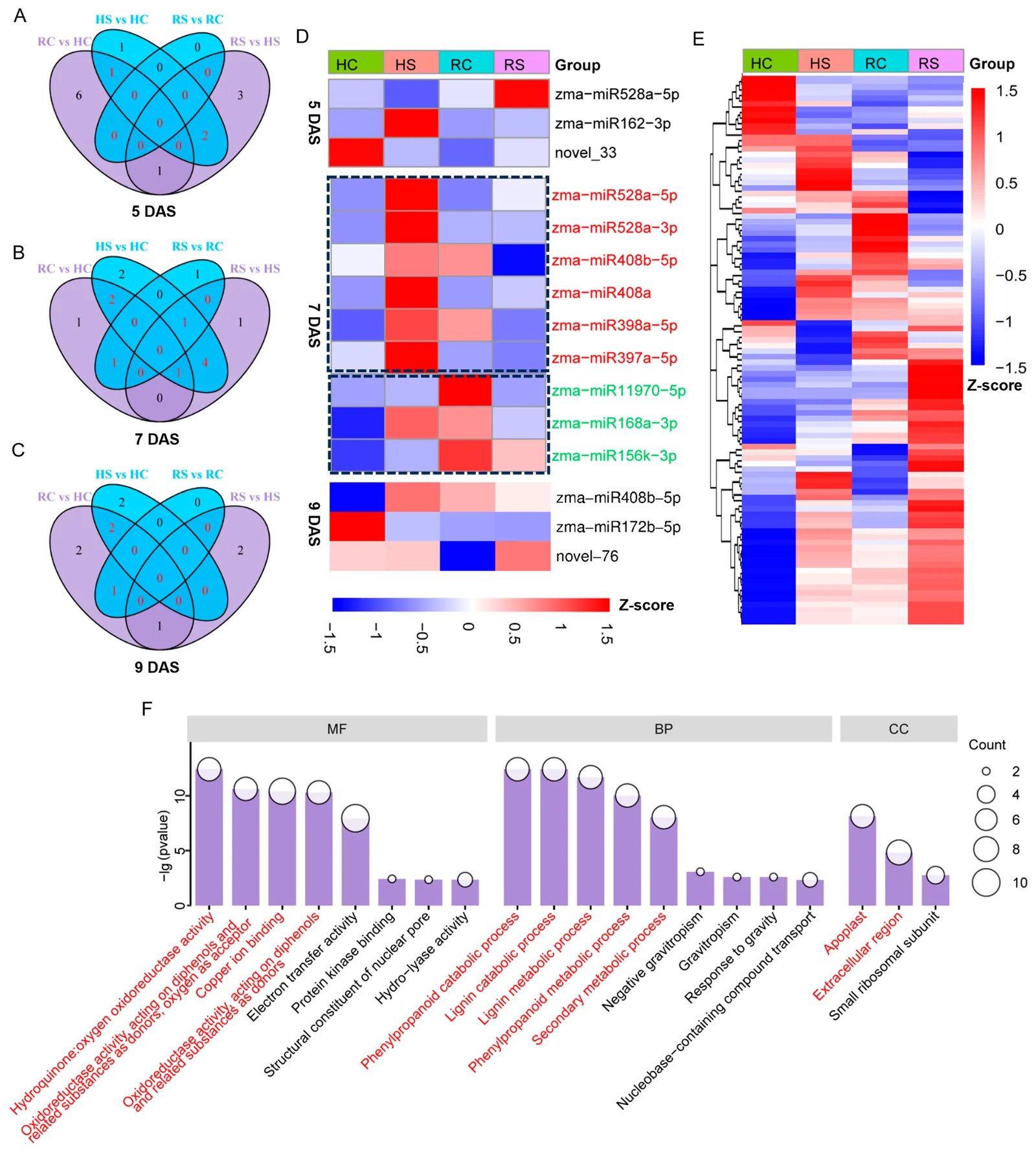
Temporal expression and predicted targets of ZmWAK-associated *SRR* miRNAs. (**A**–**C**) Venn diagrams showing differentially expressed miRNAs (DEMs) identified from the four pairwise comparisons (RC versus HC, RS versus HS, HS versus HC, and RS versus RC) at 5 (**A**), 7 (**B**), and 9 (**C**) DAS. Red numbers indicate ZmWAK-associated *SRR* miRNAs. (**D**) Expression heatmap of ZmWAK-associated *SRR* miRNAs identified at 5, 7 and 9 DAS across HC, HS, RC, and RS samples. The miRNAs shown in red are induced to be up-regulated by *S. reilianum* in HZ4, whereas those in green are induced to be down-regulated in HZ4R. (**E**) Expression heatmap of the predicted targets of ZmWAK-associated *SRR* miRNA at 7 DAS. (**F**) GO enrichment analysis of the expressed predicted targets of ZmWAK-associated *SRR* miRNA across three time points. HC, HZ4 under mock treatment; HS, HZ4 inoculated with *S. reilianum*; RC, HZ4R under mock treatment; RS, HZ4R inoculated with *S. reilianum*.

## Data Availability

The raw data presented in this study are deposited in the NCBI Sequence Read Archive (SRA). The transcriptome and microRNAome raw data bioproject accessions are PRJNA1527653 and PRJNA1528415, respectively.

## Supplementary Materials

The following supporting information can be downloaded at: https://www.mdpi.com/article/10.3390/jof12100726/s1, Table S1. ZmWAK-associated *S. reilianum* responsive (*SRR*) genes at 5 DAS; Table S2. ZmWAK-associated *S. reilianum* responsive (*SRR*) genes at 7 DAS; Table S3. ZmWAK-associated *S. reilianum* responsive (*SRR*) genes at 9 DAS; Table S4. MiRNAs detected at 5 DAS; Table S5. MiRNAs detected at 7 DAS; Table S6. MiRNAs detected at 9 DAS; Table S7. Target genes of miRNAs expressed in maize mesocotyl tissues.
